# Learning in the forest: environmental perception of Brazilian teenagers

**DOI:** 10.3389/fpsyg.2023.1046405

**Published:** 2023-07-21

**Authors:** Christiana Cabicieri Profice, Fernando Enrique Grenno, Ana Cláudia Fandi, Stela Maria Menezes, Cecília Inés Seminara, Camila Righetto Cassano

**Affiliations:** ^1^Department of Philosophy in Human and Social Sciences. Universidade Estadual de Santa Cruz, Ilhéus, Brazil; ^2^Graduate Program in Environment and Development, Universidade Estadual de Santa Cruz, Ilhéus, Brazil; ^3^Antwerp Zoo Research and Conservation Center/ Bicho do Mato Research Institute, Una, Brazil; ^4^Department of Biological Sciences. Universidade Estadual de Santa Cruz, Ilhéus, Brazil

**Keywords:** environmental perception, environmental knowledge, interpretative trail, person-nature, drawings analysis

## Abstract

The idea of separation between person and nature, accentuated by current production and consumption models, has generated unthinkable impacts, causing an unprecedented loss and degradation of the global environment. Occupying 13% of the Brazilian territory, the Atlantic Forest is the second-largest tropical rainforest on the American continent; however, it is one of the most threatened biomes in the world, with only 12% of the original cover. In this study, we consider that enabling young people to experience direct contact with nearby natural environments can positively influence their knowledge and feelings about the biodiversity that occurs there, contributing to its protection and conservation for current and future generations. In this study, we explore how teenagers (*n* = 17) aged between 13 and 17 years old describe and perceive the nearby natural environment before and after an interpretive trail in Una, Bahia, Brazil. Participants were asked to draw the Atlantic Forest with colored pencils on white paper and, based on the drawing, they answered the following questions: “What is in your drawing? and “What is happening in your drawing?,” in addition to other information such as the title of the drawing, difficulty of the activity, and sociodemographic aspects. Content analysis was used to analyze the information collected. From the drawings and responses of the participants, categories related to knowledge, experiences, and types of relationships with the visited place emerged. We count the frequency of drawing elements before and after the visit, together with a qualitative analysis of the descriptions of their feelings and meanings attributed to the visit, highlighting the different elements and their relationships. The results showed that, after the trail, the participants manifested bonds of proximity with the visited environment and the organisms protected there, evidencing expressive changes in their perceptions of the person-nature interaction, in the specific knowledge of the visited ecosystem, and in the different forms of relationship provided by the visitation itinerary.

## 1. Introduction

Nowadays, the interaction between people and nature has been approached from different perspectives. The most evident, especially during the Covid-19 pandemic but even before that, is linked to mental health, mostly for children and teenagers (Louv, 2005; Aydogdu, 2020). Recent studies allow us to state that environmental, political, and socio-economic crises constitute a real threat to children’s development and education (Hickman et al., 2021). The negative consequences of quarantine during the Covid-19 pandemic have reactivated the theme both in common sense and in the scientific field: people need nature. Beyond the direct impact on physical and mental health, studies have pointed that the isolation from nature makes people less aware and insensible to current environmental problems, such as of the loss of the planet’s biodiversity and climate emergencies, with further commitment to human well-being (Artaxo, 2020). Studies in this direction already viewed human well-being and conservation as two sides of the same coin that could not be treated independently. In fact, several initiatives seek to preserve nature for human well-being and protect other threatened beings, processes, and ecosystems. Examples of these initiatives are the practices in the field of environmental education.

The experiences in nature as a strategy for environmental awareness have been the keynote of these initiatives, with the “interpretative trails” or “interpretation trails” being one of the main instruments used. The first author to address the interpretative aspects of nature trails was Freeman Tilden (2007), for whom the interpretation is an educational activity of dignification and practical affiliation with an environment, aiming to reveal meanings of the interactivity between different physical, biological, and anthropic environmental factors (De Lima-Guimarães, 2010). Thus, interpretation is an educational instrument capable of adding value to the visitor’s experience, contributing to the formation of close relationships with the natural environment, its beings, and its processes (Ikemoto et al., 2009).

Therefore, direct contact with natural surroundings can affect how children and teenagers interact with nature, influencing their willingness to conserve biodiversity (Zhang et al., 2014; Soga and Gaston, 2016; Barthel et al., 2018). These studies also reinforce the hypothesis that experiences of this type can influence pro-environmental attitudes and behaviors during adulthood (Wells and Lekies, 2006; Collado et al., 2015; Chawla, 2020), emphasizing the importance of close experiences with natural beings and processes since childhood (Broom, 2017) to promote environmental actions. A more specific approach to changing environmental attitudes highlights the process of acculturation and the formation of a global mindset as important counterpoints to an eco-deficit culture (Vuong and Napier, 2015). In this sense, acculturation and global mindset absorbs and integrates changes, rejecting cultural aspects that are not compatible with the situation of the person or group, while breaking with traditional behaviors and practices that are inappropriate at the time, making room for the assimilation of new values, beliefs and attitudes. This global sponge mindset works proactively by gradually solving acculturation challenges in the direction of a new cultural core value centered around environmental conservation (Vuong, 2021).

Since the first research on the factors that contribute to the adoption of a new environmental paradigm from a gender perspective, a more pronounced pro-environmental tendency has always been observed in the female group (Zelezny et al., 2000), even among girls and teenagers (Corraliza et al., 2013). While there seems to be a consensus on the greater pro-environmental inclination on the part of girls and women, cultural differences and variations are also considered (Xiao and Hong, 2010), through risk perception (Xiao and McCright, 2012), and the influence of peers and parents on child environmentalism (Collado et al., 2017). In this regard, all studies involving person-environment are attentive to variations in results between genders.

In order to better access environmental perception and knowledge, drawing is a successful technique in research to identify the perception of children and teenagers (Barraza, 1999; Profice et al., 2021). In environmental studies, drawing has been used in studies on climate change (Pellier et al., 2014), basic botanical knowledge (Bartoszeck et al., 2015), perception of the natural environment, and attachment to the place (Profice et al., 2015) and the concept of biological reserve and nature conservation (Eckert et al., 2017), or even on ways of interacting with nature (Bolzan-de-Campos et al., 2018; Grenno et al., 2021). Initiatives and programs of education and environmental awareness with children and teenagers also use drawings to evaluate their activities, although in a less structured and controlled way than in the academic field, both in their collection and in the interpretation of their results. The subject presented here concerns the perception and knowledge that young people and children in rural areas have about the Atlantic Forest, based on an intervention carried out in 2018. Using drawing as a research tool, we explore how teenagers aged between 13 and 17 years old describe and perceive the nearby natural environment before and after an interpretive trail in Una, Bahia, Brazil.

## 2. Materials and methods

### 2.1. BioBrasil and environmental awareness

BioBrasil is a conservation project developed by the Center for Research and Conservation at the Zoological Society of Antwerp, in partnership with Bicho do Mato Research Institute, focusing on the golden-headed lion tamarin (*Leontopithecus chrysomelas*), an endemic and endangered primate species in southern Bahia. In its environmental education program, BioBrasil coordinates actions aimed at raising environmental awareness, environmental education, agroecology, and environmental communication to involve the local community in the forefront of the conservation of the golden-headed lion tamarin and its habitat. One of its strategies consists of an activity that begins with an interpretive nature trail, as well as other interactive experiences in more anthropized environments, as detailed further on.

In this context, it was possible to build a common goal for the academy and the environmental education program capable of meeting the expectations of the research groups involved, while also providing the BioBrasil team with an assessment of the impacts of nature experiences on the participants’ environmental knowledge and perception. The main objective of this work was to quantitatively and qualitatively evaluate the impacts of an environmental awareness activity in nature on the participants’ knowledge and perception of the environment. In the present study, we used drawing as a data collection tool to visualize the effect of the experience, addressing the following question: to what extent do experiences in interpretive trails and planned natural spaces alter the knowledge and perception of nature among teenagers in rural areas?

### 2.2. Study area

**Figure 1:**
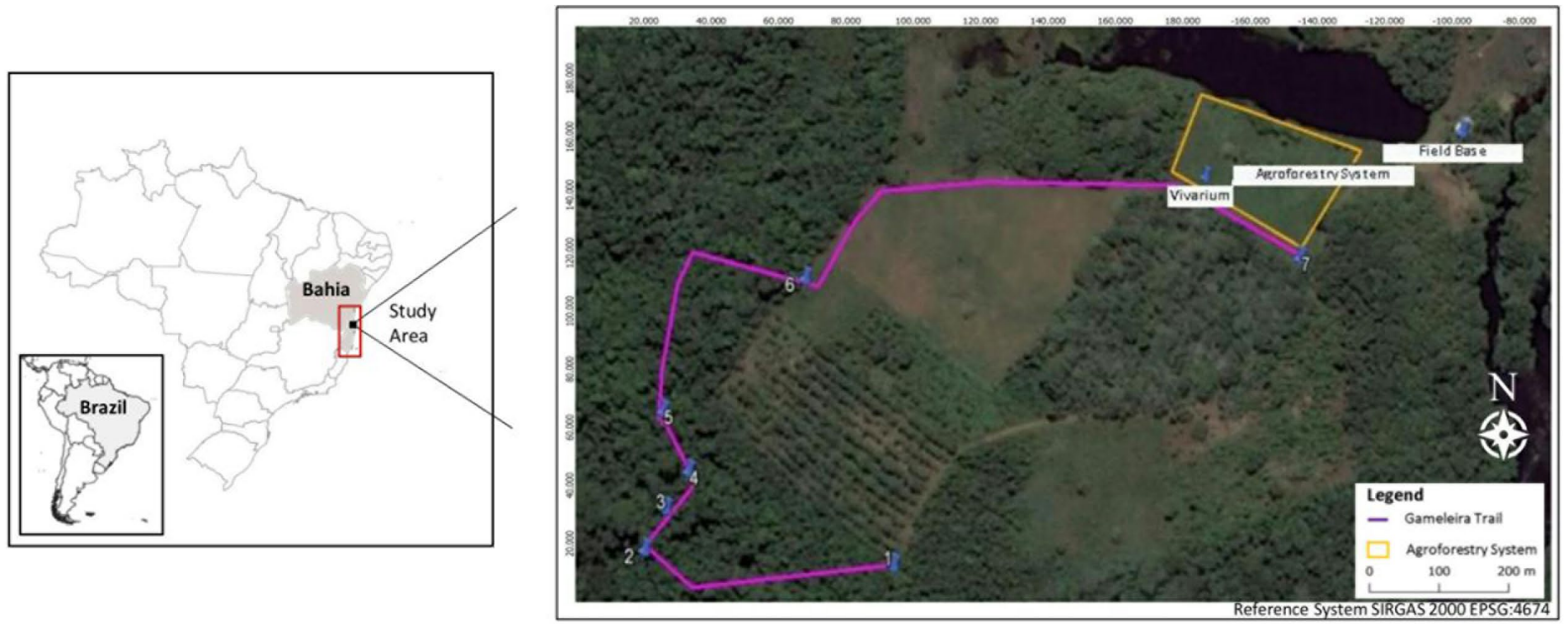
Location and route of the Gameleira Interpretive Trail located at Fazenda Santo Antônio, district of Colônia de Una, municipality of Una, state of Bahia, Brazil.

The Gameleira interpretive trail is one of the environmental awareness and perception tools used by BioBrasil to disseminate information about the Atlantic Forest and the golden-headed lion tamarin. The trail is located at Fazenda Santo Antônio, a private family-owned rural property located in the district of Colônia de Una, municipality of Una, State of Bahia, Brazil. In this area, BioBrasil also develops its research activities by monitoring 4 groups of golden-headed lion tamarins. The farm area is 1.6 miles long, with predominantly Dense Ombrophilous Forest vegetation in secondary, medium, and advanced stages of regeneration (Figure 1).

**Figure 2:**
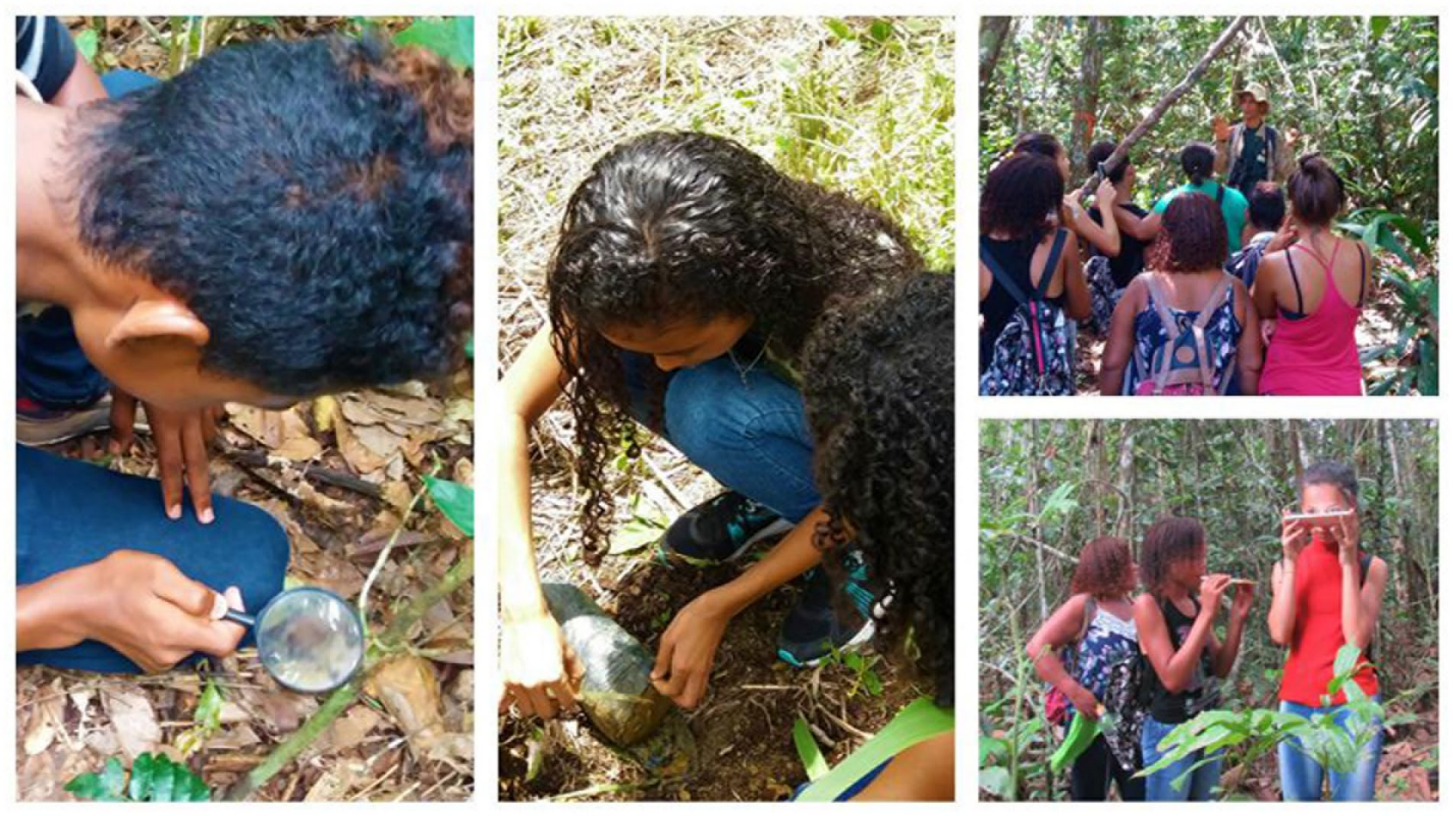
Participants during the interpretive visit to the Gameleira Trail.

The trail has 7 interpretation points to observe the difference in vegetation structure, ecosystem services, presentation of some species of flora, and interaction of fauna and flora. Thus, point 1 is used to monitor groups of golden-headed lion tamarins and their interaction with the ecosystem’s fauna and flora. Point 2 focuses on the perception of flora species and how they interact with the environment, showing characteristics of the climate and soil. Point 3 shows the constant transformation of the forest and its structure, while point 4 shows the importance of fauna for forest recovery (e.g., seed dispersal). At point 5 (the highest point of the trail), there is a centenary-old Gameleira with a hollow of the tamarin, where participants can reflect and observe the forest canopy and perceive climate change (Figure 2). In terms of the forest and its life and decomposition processes, point 6 invites participants to observe details of the forest floor through magnifying glasses. Finally, point 7 represents “The Celebration of Life,” a moment of contemplation, gratitude, and honor of the forest, remembering and re-signifying the path to reach there. The activity ends with a visit to the seedling nursery of native Atlantic Forest species, where participants are invited to plant a tree (seedling) in the agroforestry system implemented on the farm. Agroforestry has been adopted as a strategy that associates forest conservation with food production in diversified cultures (Nair, 2011).

### 2.3. Participants

The sample was composed of 17, teenagers aged between 13 and 17 years (Mean = 13.88; Standard Deviation = 0.96; 52.94% girls and 47.05% boys) eighth-grade students from Colégio Municipal Alice Fuchs de Almeida, in the city of Una (Bahia, Brazil). This study was approved by the Research Ethics Committee of the Universidade Estadual de Santa Cruz, Bahia, Brazil (C.A.E N° 01517618.8.0000.5526).

### 2.4. Research procedures

The teenagers completed the interpretive Gameleira Trail (Figure 2), guided by educators from the BioBrasil project. Before starting the trail, they watched a brief presentation about the objectives and activities developed by the project in the region, and received instructions on the day’s activity, but without details of the experience itself. Moreover, the guide in charge presented information about the monitoring work carried out by BioBrasil with groups of golden-headed lion tamarin (*Leontopithecus chrysomelas*). The Gameleira Trail is located in an area of Atlantic Forest, close to the Una Biological Reserve. This forest remnant cut by the trail concentrates a great biological richness.

In order to deepen the experience, the participants were accompanied by research assistants to learn more about the work of monitoring the lion tamarins, observe the species in the wild, and handle the radio telemetry equipment to find the animals in the forest. After the two activities (i.e., the trail itself and accompanying the monitoring of lion tamarins in the wild), participants were invited to share their experience on the banks of the Aliança River, while continuing contemplation of the environment and ending with a swim in the river.

On days and times previously coordinated by school authorities, the participants were invited to draw the Atlantic Forest with colored pencils on a square sheet of white paper (21 × 21 cm) in the classroom, 1 week before the trail. Subsequently, each participant was asked to answer individually and orally a semi-structured questionnaire with open and closed-ended questions to obtain sociodemographic information (name, age, gender) and information about the drawing, namely “What did you draw?,” “What is in your drawing?,” “What is happening in your drawing?,” “Is there any relation/function between the drawing elements?,” “Did you find it easy or difficult to draw?”; “Is there anything else you would like to say about your drawing?” and “If you had to put a title on it, what would it be?” to understand the elements, interactions, and meanings through the eyes and voice of the author. This procedure was performed twice, 7 days before and 10 days after the experience.

### 2.5. Classification of the elements represented in the drawings

**Figure 3:**
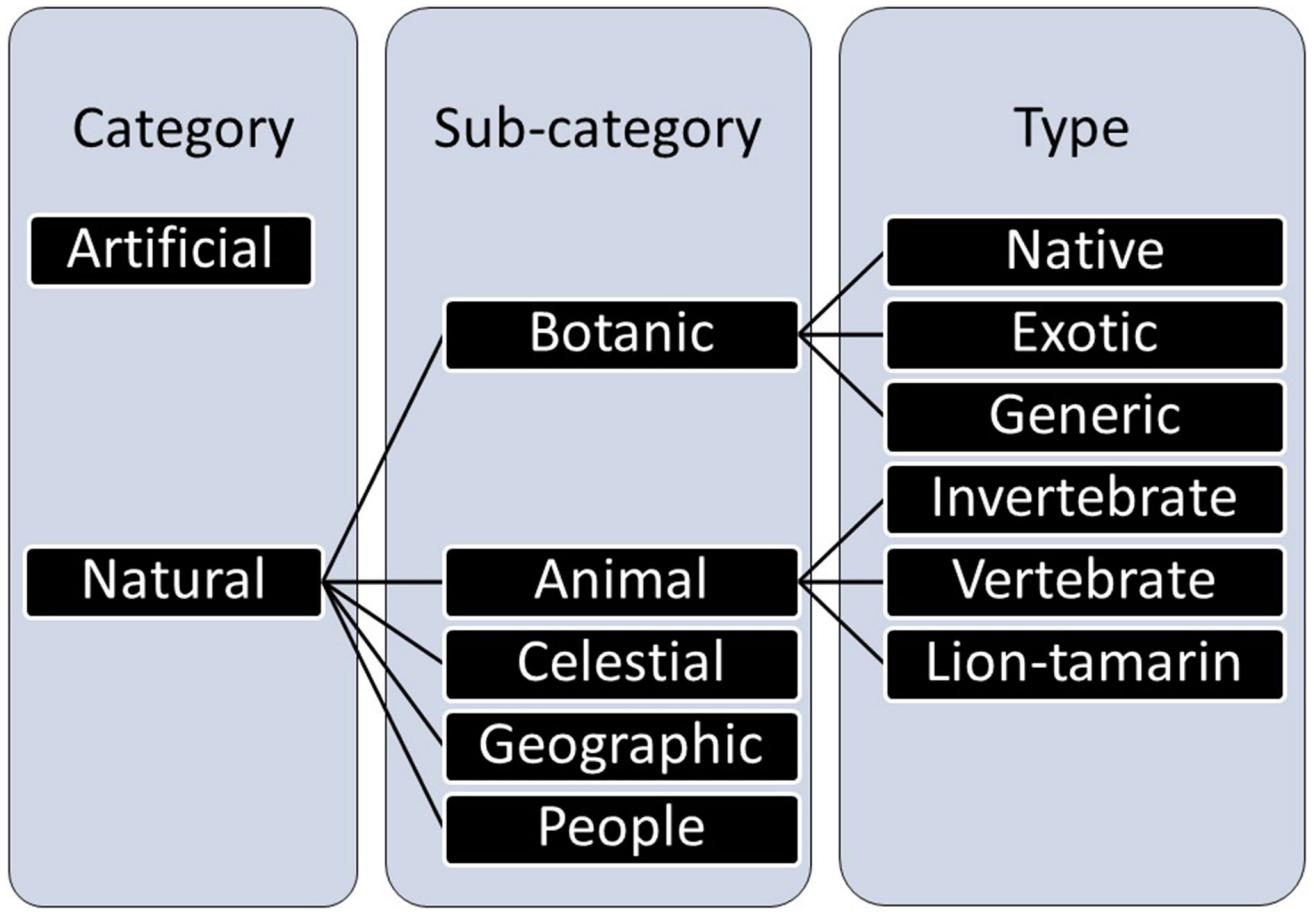
Classification of the elements represented in the drawings into categories, sub-categories, and types for quantitative analysis.

The elements in the drawings were classified following the categories suggested by Grenno et al. (2021) and Profice et al. (2015), with adaptations. Those categories were validated by a panel of judges’ techniques with scientists from biology, anthropology, and psychology, and intensively discussed to overcome disagreements before the content analysis. Thus, we first classified the drawing elements into two major categories: natural elements and artificial elements (constructions and structures). The broad category of natural elements was divided into five subcategories: botanical elements, animals, people, geographic elements (e.g., river, mountain, and sea), and celestial (e.g., cloud, sun, or rain). Finally, the botanical and animal elements of the non-human category were subdivided into types: (i) generic plants, (ii) exotic plants, (iii) native plants; (iv) vertebrate animals, (v) invertebrates animals and (vi) lion tamarin (Figure 3). The lion tamarin was considered separately from the other vertebrates, as it is the focus species of the BioBrasil project on which the students received more information, and which can be observed during the interpretative trail. For each drawing, the number of elements was quantified in each of the categories, subcategories, and types.

### 2.6. Quantitative analysis of drawings

Quantitative analyses were conducted to identify differences in groups of elements (category, sub-category, and type) drawn by different genders (boys vs. girls) and between the two moments: before and after the experience on the Gameleira Trail. We identified the groups of elements represented in at least 5 drawings (>14% of the drawings). We counted the total number of elements represented in each drawing and calculated the median and interquartile range for: (1) boys and girls and (2) before and after the experience (Table 1).

**Table 1:** Total number of elements (N), minimum, quartiles (Q1, Q2 and Q3) and maximum of elements per drawing, and number of drawings with representations in each category, sub-category, and type.

Category	Elements														Drawing	
Sub-category	Before							After							Before	After
Type	*N*	Min	Q1	Q2	Q3	Max	IQR	*N*	Min	Q1	Q2	Q3	Max	IQR	*N*	*N*
Artificial	3	0	0	0	0	1	0	23	0	0	1	3	5	3	3	9
Natural	326	5	13	17	26	31	3	370	9	16	19	25	68	3	17	17
Botanical	122	2	3	4	12	24	3	206	2	6	10	13	61	4	17	17
Native	5	0	0	0	0	4	0	22	0	0	0	1	8	1	2	5
Exotic	15	0	0	1	1	3	1	16	0	0	0	2	4	2	9	6
Generic	102	1	2	3	11	24	9	168	0	4	5	10	60	6	17	16
Animal	136	1	3	7	9	25	3	96	0	2	6	7	15	3	17	14
Invertebrate	4	0	0	0	0	4	0	13	0	0	0	0	10	0	1	4
Vertebrate	125	1	3	7	9	23	6	63	0	1	4	4	12	3	17	14
Lion tamarin	7	0	0	0	0	3	0	20	0	0	1	1	4	1	4	12
People	5	0	0	0	0	4	0	4	0	0	0	0	2	0	2	3
Celestial	43	0	1	3	4	6	3	28	0	0	1	2	13	2	13	10
Geographic	20	0	1	1	1	5	0	36	0	0	0	1	16	1	14	8
Total general	329							393							17	17

*Rightmost column represents the number of drawings containing each category and sub-category.

### 2.7. Qualitative analysis of drawings

Following the steps proposed by Bardin (2011), namely exploration of the material, treatment of inference, and interpretation of results, the qualitative analysis was oriented towards the interpretation and comparison of the meanings given both to the drawing activity and to the perception and aesthetics of nature before and after the experience. Consequently, the data were tabulated after a thorough examination of the material (drawings and comments) to build overviews that allowed a problematization of the theme addressed, especially the relevance of the experience for the articulation between the cognitive and the affective, both for the expansion of previous knowledge and for the aesthetic expressions of the portrayed nature.

## 3. Results

### 3.1. Quantitative results

Altogether, 722 elements interpreted in the drawings of the 17 participants were quantified: 329 elements were represented before, and 393 elements were represented after the experience (Table 1). All results are reported as the total number of elements (per category, sub-category, and type) represented in each drawing before and after the experience. The information regarding the total number of elements drawn by both boys and girls (before/after) will be presented in a narrative format. Median (Q2) and interquartile range for each category, sub-category and type of element before and after the experience are presented in Table 1. For more information about the median and interquartile range by gender see Supplementary Table 1.

### 3.2. Categories, subcategories, and types

**Figure 4:**
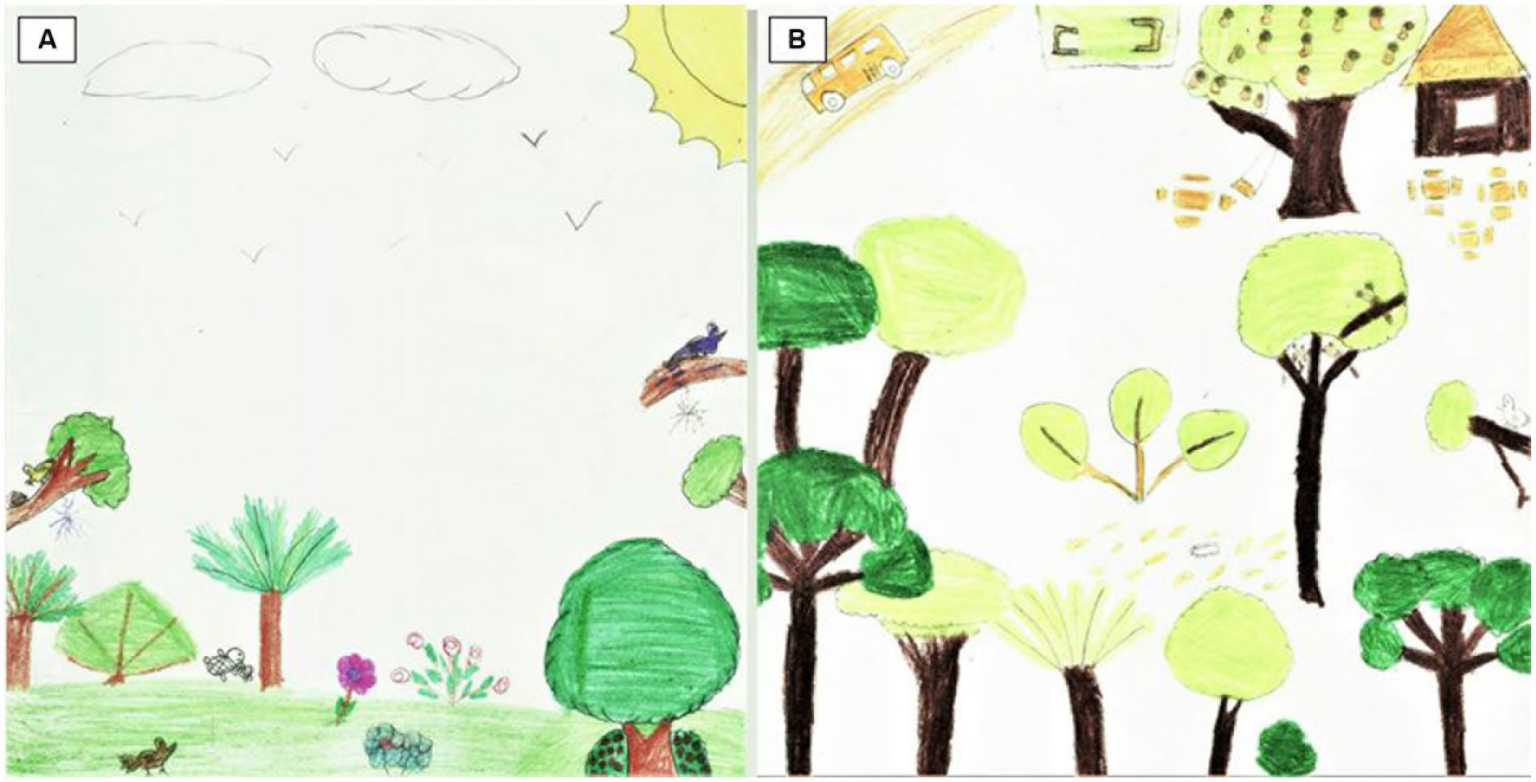
Beatriz (13 years old). Before the visit **(A)**: “The Free Farm.” After the visit **(B)**: “Knowing the lion tamarin.”

The major categories of natural and artificial elements comprised 696 and 26 items, respectively. In the first, most of the elements represented were botanicals and animals. In the post-visit drawings, aspects of the experience were represented, such as the trail itself or, even, the swing and soccer field, which were present in the recreational space used at the end of the trail (Figure 4). Considering all the elements drawn by each participant, before and after the visit (*N* = 34), 326 natural elements were represented before, and 370 after the experience. The artificial elements were represented 3 and 23 before and after the experience, respectively (see Table 1). Regarding the gender of the participants, 141 and 185 natural elements were represented, respectively, by boys and girls before the experience, and 160 and 210 after the experience. Corresponding numbers for artificial elements were 3 and 0 before the experience and 11 and 12 after the experience.

The sub-categories of botanical and animal elements were the most represented, with 328 and 232 representations, respectively. These were followed by the sub-categories of celestial, geographic, and people elements, with 71, 56, and 9 representations. Regarding the botanical elements, 122 and 206 were represented, respectively, before and after the experience, and the frequency of animal representations was 136 before, and 96 after. Among the abiotic elements (geographic and celestial), rivers, lagoons, and the sea stood out. In total, 20 geographic elements were represented before and 36 after the experience, while celestial elements were represented 43 before and 28 after the experience (Table 1). People were poorly represented in both moments, with 9 representations in 5 drawings only. Regarding the gender of the participants, 28 and 94 botanical elements were represented, respectively, by boys and girls before the experience, and 58 and 148 after. Corresponding numbers for animal elements were 71 and 65 before and 48 and 48 after the experience.

Among the botanical elements, students mainly represented generic plants (270 representations in 33 drawings), followed by exotic plants (especially fruits, which had 31 representations in 15 drawings) and native plants (27 representations in 7 drawings). In total, 102 generic botanical elements were represented before and 168 after the experience, while the representation of exotic botanical elements was 15 and 16 after. Regarding the gender of participants, 17 and 85 generic botanical elements were represented, respectively, by boys and girls before, and 34 and 134 after the experience. Corresponding numbers for exotic botanical elements were: 7 and 8 before the experience and 10 and 6 after the experience.

**Figure 5:**
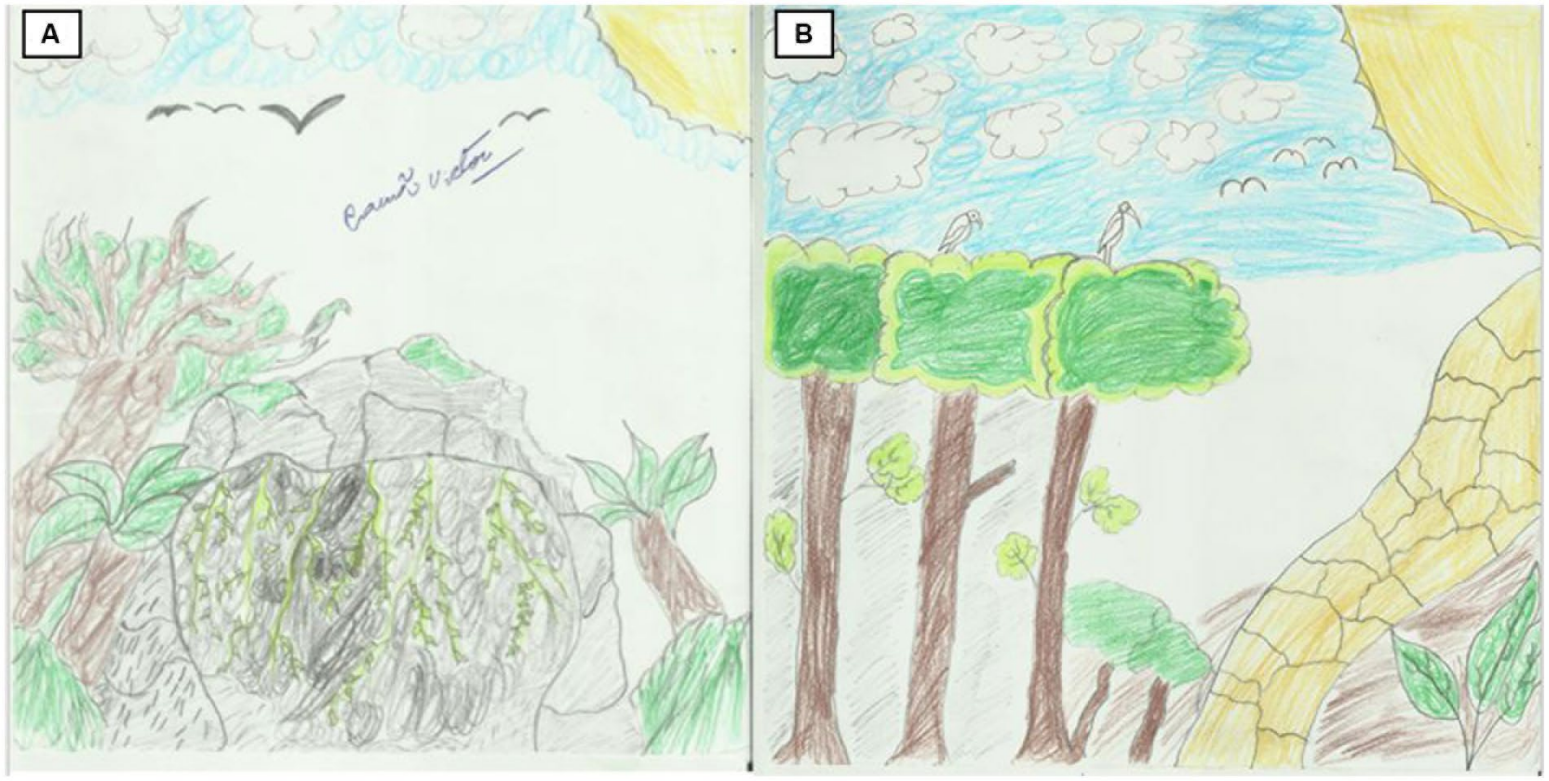
Cauã (14 years old). Before the visit **(A)**: “The Unknown Cave.” After the visit **(B)**: “Pine Forest.”

**Figure 6:**
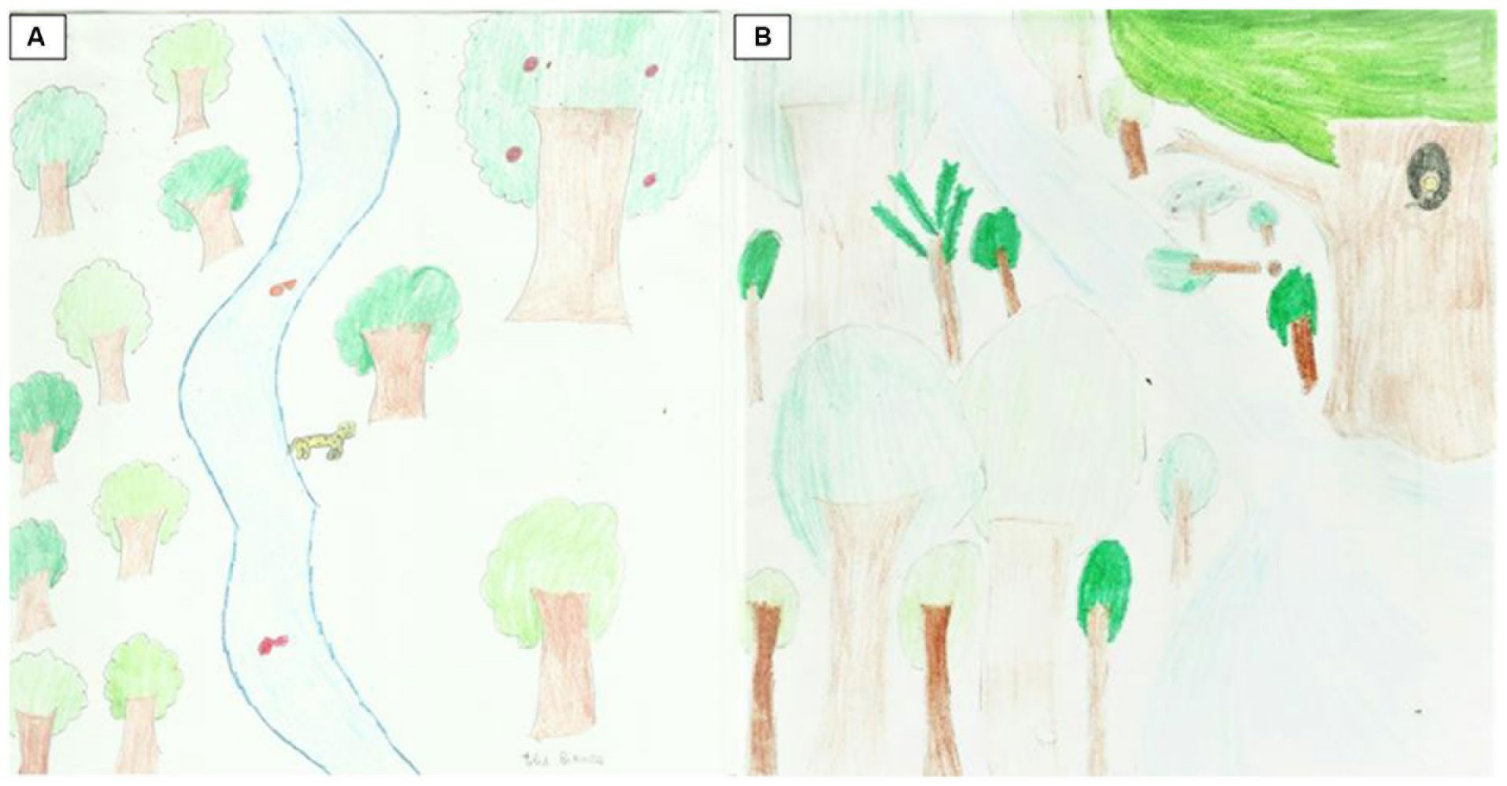
Ellis (13 years old). Before the visit **(A)**: “Atlantic Forest.” After the visit **(B)**: “Diversity of the Atlantic Forest.”

In the animal elements, vertebrates, in general, were more frequent (188 representations in 31 drawings), followed by the lion tamarin (27 representations in 16 drawings) and invertebrates (17 representations in 5 drawings). The most represented vertebrates were birds, with many generic representations (line style), although there was a diversity of species such as toucans, parrots, and herons (Figure 5). Mammals were represented exclusively by the lion tamarin, jaguar (2 drawings), and domestic dog (1 drawing). In total, 125 vertebrates were represented before the experience and 63 after the experience. Lion tamarin was represented: 7 before the experience, and 20 after the experience (see Table 1; Figure 6). Regarding the gender of the participants, 69 and 56 vertebrates were represented, respectively, by boys and girls before the experience, and 34 and 29 after the experience. Corresponding numbers for lion tamarin were 2 and 5 before the experience, and 4 and 16 after the experience.

### 3.3. Qualitative results

This section provides an overview of the drawings made before and after the trail and time with the research assistants, with some examples and statements that guided our reflections. The first constant that stands out in the drawings and statements of the participants is a feeling of greater ease in drawing the Atlantic Forest after completing the trail. Many participants stated that the first drawing was difficult to execute because they had the self-perception of not knowing the subject, despite living in this environment and frequenting the woods in their daily lives. Notably, the experience of the trail was fundamental for the participants to consider what they had learned about the subject based on the newly acquired knowledge and affections and the knowledge they already had.

“It was easier because, before the trail, I did not know what to draw, after the trail, things are clearer in my mind” (Emily, 13 years old).

“Easier than before because I had drawn thin trees and not thick ones like I saw in the woods” (Yami, 13 years old?).

We also identified an expressive difference both in the knowledge of the functioning of the Atlantic Forest and its configuration and aesthetics, with landscapes where the color of the drawn elements is mostly green. In the drawings by Elis (13 years old), we can observe a change in the structure of the forest between the activities before and after the trail (Figure 6). She found the first drawing difficult since she believed her knowledge of the Atlantic Forest was almost non-existent. In the drawn scene, we observe a jaguar looking for food in a very open area and an apple tree among the trees that are far from each other. It is worth mentioning that the apple tree is not found in this region. For this teenager, it was easier to draw a forest after the trail. In the second scene, the forest is more diversified and closed, with only native plant species such as the gameleira and the presence of the golden-headed lion tamarin. A fallen tree indicates deforestation and its risk to animals. She declared that humans are absent because they harm the forest and named her drawing “Atlantic Forest Diversity.”

Likewise, when interviewed after the visit and after her second drawing, Emily (13 years old) stated, “It was easier because, before the trail, I did not know what to draw, after the trail things are clearer in my mind.” Cauã, 14 years old, named his first drawing “The Unknown Cave” and declared that it was difficult and that he could not draw properly because he does not know how to draw well. In the image, we see a cave with bushes on top and an apple tree together with other local animal species such as toucans and parakeets. A change of landscape is perceptible between the first and second drawings, which is more alive, green, organized, and careful than in the previous drawing. He declared, “This time it was easy,” and in his “Pine Forest,” we find coconut trees and branches, the herons looking for the flock to migrate, and the jaguar following the wild boar in the forest (Figure 5).

**Figure 7:**
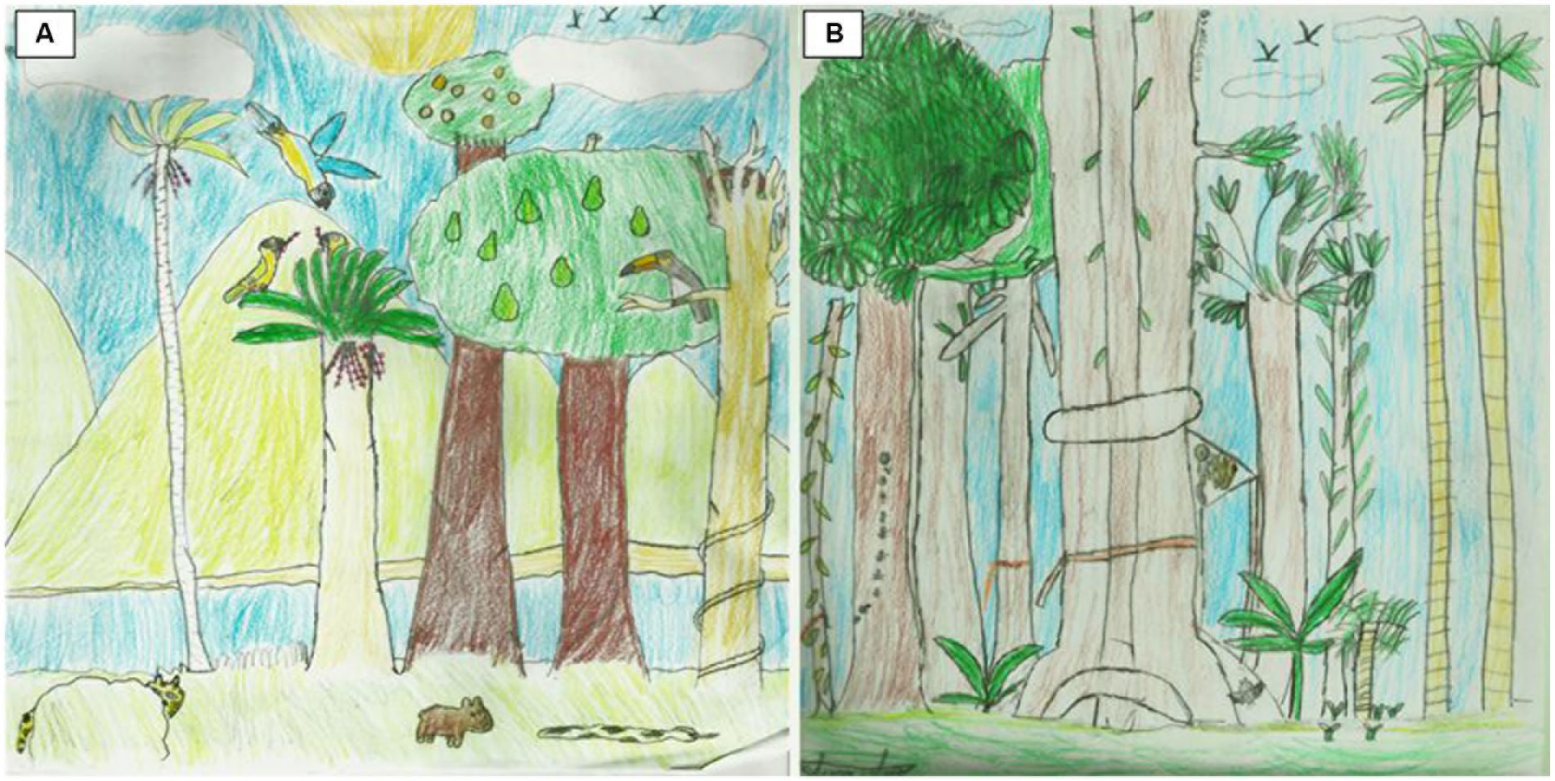
Jaivan (14 years old). Before the visit **(A)**: “The Snack Time.” After the visit **(B)**: “Fauna and Flora of BioBrasil.”

Jaivan, aged 14, made skilled drawings (Figure 7), the first of which he portrayed wild animals and local trees such as the avocado tree. He commented that he did not draw the blue macaw because it is from another biome. In “The Snack Time,” he sought to produce a scene in which “everyone was doing something.” He said the first drawing was a little difficult because it is full of details and things to paint. The drawing after experiencing the trail is also very beautiful, with more emphasis on vegetation, and the presence of the golden-headed lion tamarin, ants, and bats. Although he declared that he liked the result, he found the execution difficult again due to the many details he saw in the trees. He named his drawing “Fauna and Flora of BioBrasil.” Here the teenager started from a well-structured knowledge and expanded it, thus demonstrating his skill in graphic production and focusing on its elements and retraction.

## 4. Discussion

Our results showed some changes in the way teenagers perceive and get to know the Atlantic Forest, reflecting the participants’ connection with the visit and the importance of direct experiences with aesthetic manifestations of nature that highlight the lived experience, with drawings more in tune with the local reality.

In the representations and frequencies by each category, the results highlight an Atlantic Forest inhabited mainly by natural botanical and animal elements and with little human presence, evidencing a mostly common perception of nature among children and teenagers already pointed out in other studies (Dai, 2017; Bolzan-de-Campos et al., 2018; Grenno et al., 2021). However, the higher presence of constructed elements in the post-visit drawings seems to contrast with their previous perception, perhaps due to the influence of media, films, or cartoons (Barraza, 1999). Thus, we observed that most of the elaborations after the experience are illustrative of the visited place, revealing interconnections between the affective-sensory and the lived experience (Barthel et al., 2018).

Although the great frequency of botanical and animal elements may have been influenced by the request to the participants to draw “the Atlantic Forest,” the fact that they accompany the activities carried out by the community in the forest in their daily lives contributes to their repertoire. In this regard, although we have not observed an increase in the identification of native botanical elements of local origin, the increase in the total number of botanical elements after the experience suggests some implications for teenager’s conservation attitude, according to other studies which show a concern of adolescents with environmental problems (Barros and Pinheiro, 2020; Keith et al., 2022). In addition, the post-visit drawings show a view that is relatively more consistent with the visited place, where floristic exuberance and diversity are predominant (Figure 7), stimulated and expanded by the activities proposed during the experience, which included, for example, planting a seedling in the agroforestry system (Figure 2).

As previously mentioned, in addition to observing an increase in generic botanical elements, the presence of vertebrates increased in the drawings after the visit. These vertebrates, in agreement with other studies (Profice et al., 2015; Grenno et al., 2021), are mostly represented by birds, which were more than half of the simple line drawing type, suggesting a low difficulty in the execution and, consequently, relative success in the elaboration.

In contrast, the fact that mammals are represented almost exclusively by the golden-headed lion tamarin, and its frequency seems to increase considerably in the drawings after the experience, shows the assimilation of the natural elements when they are experienced through direct contact with the environment (Kellert, 1993, 2002; Payne, 1998). Additionally, the importance of the two educators during the activity is highlighted to raise awareness among the participants, enriching and amplifying the experience. Activities in interpretative trails are powerful educational tools (Ikemoto et al., 2009), but environmental educators should carefully plan what to present and emphasize to impact youth perceptions, considering what are key learnings and understandings that are important to share with the participants.

Although it was not our main intention, we could not fail to observe the gender differences related to the expressive increase in the representation of the golden-headed lion tamarin in the drawings and comments after the visit, reinforcing the idea, already pointed out in previous studies, that suggests greater environmental awareness among girls than boys (Ramstetter and Habersack, 2020).

The fact that some participants expressed their difficulty in representing what they wanted through the language of drawing can be attributed to the few opportunities for artistic expression offered by conventional schools, as well as the age group characterized by a lack of interest in drawing as a form of expression (Profice et al., 2021). However, the results also reinforce the relevance of the use of drawings as a research tool with children and young people, in line with previous studies (Barraza, 1999; Profice et al., 2015; Bolzan-de-Campos et al., 2018), and the importance of accompanying written or oral descriptions (Bland, 2018).

Regarding issues of awareness and expansion of knowledge of the trail (differences and vegetation structure, ecosystem services, species of native flora, and characteristics of the climate and soil), although our analysis was not carried out to verify whether all of them changed, our results indicate a positive impact regarding these factors.

## 5. Final considerations

One of the limitations of this research was that the drawing and questionnaire activities were carried out 10 days after the visit, which did not allow a long-term impact perspective. In this regard, we encourage future studies to use the drawing activities and questionnaires on different occasions — 3, 6 months, or 1 year after the trail — and to determine if perceptions remain altered by the experience.

Despite living in and seeing the forest daily, its resignification, which was the objective of the trail, can generate a new environmental perception. The visit to the trail can improve and consolidate prior knowledge, and the experience can expand this knowledge. Living in nature, besides increasing knowledge and perception, contributes to our ability to represent it. In this regard, the self-perceived lack of mastery of graphic skills may indicate that the arts and drawing are not common practices in schools and homes.

In summary, our results show that, regardless of gender, experiences of interpretive trails in direct contact with the natural environment can be a powerful instrument for the consolidation of knowledge and close links with the visited environment and the beings protected there. We also observed the importance of this type of contact for triggering aesthetic manifestations of nature, in which the lived experience stands out, with drawings that are more in line with the local reality. Our research supports the idea that children are aware of environmental problems and demand guidance from the adult and institutional world for their solution. It is imperative to meet this demand towards environmental commitment and allow the new generations to do better than the previous ones (Sousa et al., 2021). In terms of open-mindedness, our research points to childhood as the period when human plasticity to overcome beliefs and values and adopt new attitudes towards reality is at its fullest, that is, it is when acculturation encounters less resistance (Vuong and Napier, 2015).

Beyond the resignification and consolidation of knowledge and perception of the visited environment and the beings protected there, the pleasurable experiences provided by interpretative trails are likely to enhance physical and mental health. Altogether, the benefits of this type of activity in the open environment have a great potential to enhance human well-being and increase nature conservancy. Finally, we would like to highlight that the combination of research objectives between academia and environmental awareness programs points to a production of knowledge based on the current reality that can support future actions with the communities involved.

## Data availability statement

The raw data supporting the conclusions of this article will be made available by the authors, without undue reservation.

## Ethics statement

The studies involving human participants were reviewed and approved by Comitê de Ética em Pesquisa-Universidade Estadual de Santa Cruz/UESC. Written informed consent to participate in this study was provided by the participants’ legal guardian/next of kin. Written informed consent was obtained from the individual(s), and minor(s)’ legal guardian/next of kin, for the publication of any potentially identifiable images or data included in this article.

## Author contributions

AF, SM, CP, and CS conducted the fieldwork. FG analyzed the data and wrote most of the manuscript. CC participated in fieldwork logistics and contributed to writing and discussion. All authors contributed to the article and approved the submitted version.

## Funding

This study was supported by Antwerp Zoo Research and Conservation Center/ Bicho do Mato Research Institute, UESC, and CAPES.

## Conflict of interest

The authors declare that the research was conducted in the absence of any commercial or financial relationships that could be construed as a potential conflict of interest.

## Publisher’s note

All claims expressed in this article are solely those of the authors and do not necessarily represent those of their affiliated organizations, or those of the publisher, the editors and the reviewers. Any product that may be evaluated in this article, or claim that may be made by its manufacturer, is not guaranteed or endorsed by the publisher.

## References

[ref1] Artaxo P. (2020). As três emergências que nossa sociedade enfrenta: saúde, biodiversidade e mudanças climáticas. Estud. Av. 34, 53–66. doi: 10.1590/s0103-4014.2020.34100.005

[ref2] Aydogdu F. A. L. (2020). Saúde mental das crianças durante a pandemia causada pelo novo coronavírus: revisão integrativa. J. Health NPEPS 5:e-4891. doi: 10.30681/252610104891

[ref3] Bardin L (2011). Análise de Conteúdo. São Paulo: Edições 70, 229p.

[ref4] Barraza L. (1999). Children’s drawings about the environment. Environ. Edu. Res. 5, 49–66. doi: 10.1080/1350462990050103

[ref5] Barros H. Pinheiro J. (2020). Climate change perception by adolescents: reflections on sustainable lifestyle, local impacts and optimism bias. Psyecology 11, 260–283. doi: 10.1080/21711976.2020.1728654

[ref6] Barthel S. Belton S. Raymond C. M. Giusti M. (2018). Fostering children’s connection to nature through authentic situations: the case of saving salamanders at school. Front. Psychol. 9, 1–15. doi: 10.3389/fpsyg.2018.00928 29937747 PMC6002744

[ref7] Bartoszeck A. B. Cosmo C. R. Dasilva B. R. Tunnicliffe S. D. (2015). Concepts of plants held by young Brazilian children: an exploratory study. Eur. J. Edu. Res. 4, 105–117. doi: 10.12973/eu-jer.4.3.105

[ref8] Bland D. (2018). Using drawing in research with children: lessons from practice. Int. J. Res. Method Edu. 41, 342–352. doi: 10.1080/1743727X.2017.1307957

[ref9] Bolzan-de-Campos C. Fedrizzi B. Santos-Almeida C. R. (2018). How do children from different settings perceive and define nature? A qualitative study conducted with children from southern Brazil. Psyecology 9, 177–203. doi: 10.1080/21711976.2018.1432526

[ref10] Broom C. (2017). Exploring the relations between childhood experiences in nature and young adults’ environmental attitudes and behaviours. Aust. J. Environ. Educ. 33, 34–47. doi: 10.1017/aee.2017.1

[ref11] Chawla L. (2020). Childhood nature connection and constructive hope: a review of research on connecting with nature and coping with environmental loss. People Nat. 2, 619–642. doi: 10.1002/pan3.10128

[ref12] Collado S. Corraliza J. A. Staats H. Ruiz M. (2015). Effect of frequency and mode of contact with nature on children’s self-reported ecological behaviors. J. Environ. Psychol. 41, 65–73. doi: 10.1016/j.jenvp.2014.11.001

[ref13] Collado S. Evans G. W. Sorrel M. A. (2017). The role of parents and best friends in children’s pro-environmentalism: differences according to age and gender. J. Environ. Psychol. 54, 27–37. doi: 10.1016/j.jenvp.2017.09.007

[ref14] Corraliza J. A. Collado S. Bethelmy L. (2013). Spanish version of the new ecological paradigm scale for children. Span. J. Psychol. 16, 1–8. doi: 10.1017/sjp.2013.46 23866221

[ref15] Dai A. (2017). “Learning from children’s drawings of nature” in Drawing for Science Education: An International Perspective. ed. Katz P. (Rotterdam: SensePublishers), 73–86.

[ref16] De Lima-Guimarães S. T. (2010). Trilhas Interpretativas e Vivências na Natureza : aspectos relacionados à percepção e interpretação da paisagem. Cad. Geogr. 20, 8–19. doi: 10.13140/RG.2.1.1816.9444

[ref17] Eckert N. O. S. Bonfim L. S. A. Santana R. T. S. Santos F. A. S. Faiad P. J. B. Coelho A. S. (2017). Environmental perception of rural students about the Biological Reserve of Santa Isabel, Pirambu (SE). Braz. J. Environ. Educ. 12, 43–57. doi: 10.34024/revbea.2017.v12.2237

[ref18] Grenno F. E. Martinez R. A. Profice C. C. (2021). Experience in a protected area of the Atlantic Forest changed the way children and teenagers described nature. Ecopsychology 13, 174–185. doi: 10.1089/eco.2020.0055

[ref19] Hickman C. Marks E. Pihkala P. Clayton S. Lewandowski E. Mayall E.. (2021). Climate anxiety in children and young people and their beliefs about government responses to climate change: a global survey. Lancet Planet Health. 5, 863–873. doi: 10.1016/S2542-5196(21)00278-3 34895496

[ref20] Ikemoto S. M. De Moraes M. G. Da Costa V. C. (2009). Avaliação do potencial interpretativo da trilha do Jequitibá, Parque Estadual dos Três Picos, Rio de Janeiro. Soc. Nat. 21, 271–287. doi: 10.1590/s1982-45132009000300004

[ref21] Keith R. J. Given L. M. Martin J. M. Hochuli D. F. (2022). Urban children and adolescents’ perspectives on the importance of nature. Environ. Educ. Res. 28, 1547–1563. doi: 10.1080/13504622.2022.2080810

[ref22] Kellert S. R. (1993). “The biological basis for human values of nature” in The Biophilia Hypothesis. eds. Kellert S. R. Wilson E. O. (Washington DC: Island Press), 42–69.

[ref23] Kellert S. R. (2002). “Experiencing nature: affective, cognitive, and evaluative development in children” in Children and Nature: Psychological, Sociocultural, and Evolutionary Investigations. eds. Kahn P. H. Kellert S. R. (Cambridge: MIT Press), 117–151.

[ref24] Louv R. (2005). Last Child in the Woods: Saving Our Children From Nature-Deficit Disorder. Chapel Hill, NC: Algonquin.

[ref25] Nair P. K. R. (2011). Agroforestry systems and environmental quality: introduction. J. Environ. Qual. 40, 784–790. doi: 10.2134/jeq2011.0076 21546663

[ref26] Payne P. (1998). Children’s conceptions of nature. Aust. J. Environ. Educ. 14, 19–26. doi: 10.1017/S0814062600003918

[ref27] Pellier A. S. Wells J. A. Abram N. K. Gaveau D. Meijaard E. (2014). Through the eyes of children: perceptions of environmental change in tropical forests. PLoS One 9:e103005. doi: 10.1371/journal.pone.0103005 25093658 PMC4122389

[ref28] Profice C. Grenno F. E. Menezes S. M. Montaño R. M. Amim V. (2021). Children’ s drawings as an experience – scope and limits of their revelations. Int. J. Dev. Res. 11, 43997–44005. doi: 10.37118/ijdr.20886.01.2021

[ref29] Profice C. Pinheiro J. Q. Fandi A. C. Gomes A. R. (2015). Children’s environmental perception of protected areas in the Atlantic rainforest. Psyecology 6, 328–358. doi: 10.1080/21711976.2015.1026085

[ref30] Ramstetter L. Habersack F. (2020). Do women make a difference? Analysing environmental attitudes and actions of members of the European Parliament. Environ. Politics. 29, 1063–1084. doi: 10.1080/09644016.2019.1609156

[ref31] Soga M. Gaston K. J. (2016). Extinction of experience: the loss of human-nature interactions. Front. Ecol. Environ. 14, 94–101. doi: 10.1002/fee.1225

[ref32] Sousa T. Silva T. Ramos M. (2021). What factors can influence children’s perception of forests today and in the future? Ethnobiol. Conserv. 10, 10–13. doi: 10.15451/ec2021-04-10.19-1-13

[ref33] Tilden F. (2007). Interpreting Our Heritage. Revised Edn. Chapel Hill: The University of North Carolina Press.

[ref34] Vuong Q.-H. (2021). The semiconducting principle of monetary and environmental values exchange. Econ. Bus. Lett. 10, 284–290. doi: 10.17811/ebl.10.3.2021.284-290

[ref35] Vuong Q.-H. Napier K. (2015). Acculturation and global mindsponge: an emerging market perspective. Int. J. Intercult. Relat. 49, 354–367. doi: 10.1016/j.ijintrel.2015.06.003

[ref36] Wells N. M. Lekies K. S. (2006). Nature and the life course: pathways from childhood nature experiences. Child. Youth Environ. 16, 1–24. doi: 10.1353/cye.2006.0031

[ref37] Xiao C. Hong D. (2010). Gender differences in environmental behaviors in China. Popul. Environ. 32, 88–104. doi: 10.1007/s11111-010-0115-z

[ref38] Xiao C. McCright A. M. (2012). Explaining gender differences in concern about environmental problems in the United States. Soc. Nat. Resour. 25, 1067–1084. doi: 10.1080/08941920.2011.651191

[ref39] Zelezny L. C. Chua P. P. Aldrich C. (2000). New ways of thinking about environmentalism: elaborating on gender differences in environmentalism. J. Soc. Issues 56, 443–457. doi: 10.1111/0022-4537.00177

[ref40] Zhang W. Goodale E. Chen J. (2014). How contact with nature affects children’s biophilia, biophobia and conservation attitude in China. Biol. Conserv. 177, 109–116. doi: 10.1016/j.biocon.2014.06.011

