# Supplementary material for: Learning in the forest: environmental perception of Brazilian teenagers
Source: Front Psychol. 2023 Jul 21;14:1046405. doi: 10.3389/fpsyg.2023.1046405 (PMC10403060; doi:10.3389/fpsyg.2023.1046405)
Supplement: Supplementary file 1 [file Table_1.DOCX]

**Supplementary Materials**

**Total elements drawn (N) by all participants before and after the visit (N = 34) with the minimum, quartiles (Q1, Q2, and Q3), and maximum elements drawn according to gender, represented in each category, subcategory, and type.**

|  | **Elements** | | | | | | | | | | | | | | | | | | |  | | | **Drawing** | | | | |  |
| --- | --- | --- | --- | --- | --- | --- | --- | --- | --- | --- | --- | --- | --- | --- | --- | --- | --- | --- | --- | --- | --- | --- | --- | --- | --- | --- | --- | --- |
| **CATEGORY** | **Girl** | | | | | |  | | |  | **Boy** | | | | | | | | |  | | | **Girl** | | **Boy** | | |  |
| **Sub-category** |  |  |  |  |  |  |  | | |  |  |  |  |  |  |  |  |  |  |  | | |  |  |  |  |  |  |
| **Type** | **N** | min | Q1 | Q2 | Q3 | max | |  | **N** | | | min | Q1 | Q2 | Q3 | | max | | | |  | | | **N** | **N** | | | |
| **ARTIFICIAL** | 12 | 0 | 0 | 0 | 1 | 3 | |  | 14 | | | 0 | 0 | 0 | 1 | | 5 | | | |  | | | 6 | 6 | | | |
| **NATURAL** | 395 | 10 | 14 | 18.5 | 25 | 68 | |  | 301 | | | 5 | 14 | 18 | 25 | | 30 | | | |  | | | 18 | 16 | | | |
| **Botanical** | 242 | 2 | 4 | 12 | 17 | 61 | |  | 86 | | | 2 | 3 | 4 | 7 | | 13 | | | |  | | | 18 | 16 | | | |
| **Native** | 9 | 0 | 0 | 0 | 0 | 7 | |  | 18 | | | 0 | 0 | 0 | 0.5 | | 8 | | | |  | | | 3 | 4 | | | |
| **Exotic** | 14 | 0 | 0 | 0 | 1 | 3 | |  | 17 | | | 0 | 0 | 0.5 | 1.5 | | 4 | | | |  | | | 7 | 8 | | | |
| **Generic** | 219 | 2 | 4 | 10.5 | 14 | 60 | |  | 51 | | | 0 | 2 | 3 | 4 | | 8 | | | |  | | | 18 | 15 | | | |
| **Animal** | 113 | 0 | 2 | 6 | 8 | 22 | |  | 119 | | | 0 | 3.5 | 6.5 | 9.5 | | 25 | | | |  | | | 17 | 14 | | | |
| **Invertebrate** | 7 | 0 | 0 | 0 | 0 | 4 | |  | 10 | | | 0 | 0 | 0 | 0 | | 10 | | | |  | | | 4 | 1 | | | |
| **Vertebrate** | 85 | 0 | 2 | 4 | 7 | 15 | |  | 103 | | | 0 | 3 | 5.5 | 7.5 | | 23 | | | |  | | | 17 | 14 | | | |
| **Lion tamarin** | 21 | 0 | 0 | 1 | 2 | 4 | |  | 6 | | | 0 | 0 | 0 | 1 | | 2 | | | |  | | | 11 | 5 | | | |
| **People** | 3 | 0 | 0 | 0 | 0 | 2 | |  | 6 | | | 0 | 0 | 0 | 0 | | 4 | | | |  | | | 2 | 3 | | | |
| **Celestial** | 22 | 0 | 0 | 0 | 3 | 5 | |  | 49 | | | 0 | 1 | 2 | 4 | | 13 | | | |  | | | 8 | 15 | | | |
| **Geographic** | 15 | 0 | 0 | 1 | 1 | 3 | |  | 41 | | | 0 | 0 | 1 | 1 | | 16 | | | |  | | | 12 | 10 | | | |
| **TOTAL GENERAL** | **407** |  |  |  |  |  | |  | **315** | | |  |  |  | |  | |  |  | | | **18** | | | | **16** |  |  |
| * Q2 in the table represents the median, and the rightmost column represents the number of drawings containing each category and sub-category. | | | | | | | | | | | | | | | | | | | | | | | | | | |  |  |
